# Breast myeloid sarcoma

**DOI:** 10.1002/jha2.306

**Published:** 2021-10-01

**Authors:** Adam C. Gonzalez, Felix A. Olobatuyi, Kirill A. Lyapichev

**Affiliations:** ^1^ Department of Pathology The University of Texas Medical Branch Galveston Texas USA; ^2^ HCA Houston Healthcare Medical Center Forward Pathology Solutions Houston Texas USA

A 53‐year‐old female with history of left breast lumpectomy for ductal carcinoma in situ and a phyllodes tumor presented with two new right breast masses: A 10 cm irregularly shaped mass centered along the 10:00 axis, 8 cm from the nipple, and a 4.3 cm irregularly shaped mass along the 3:00 axis, 5 cm from the nipple.

**FIGURE 1:**
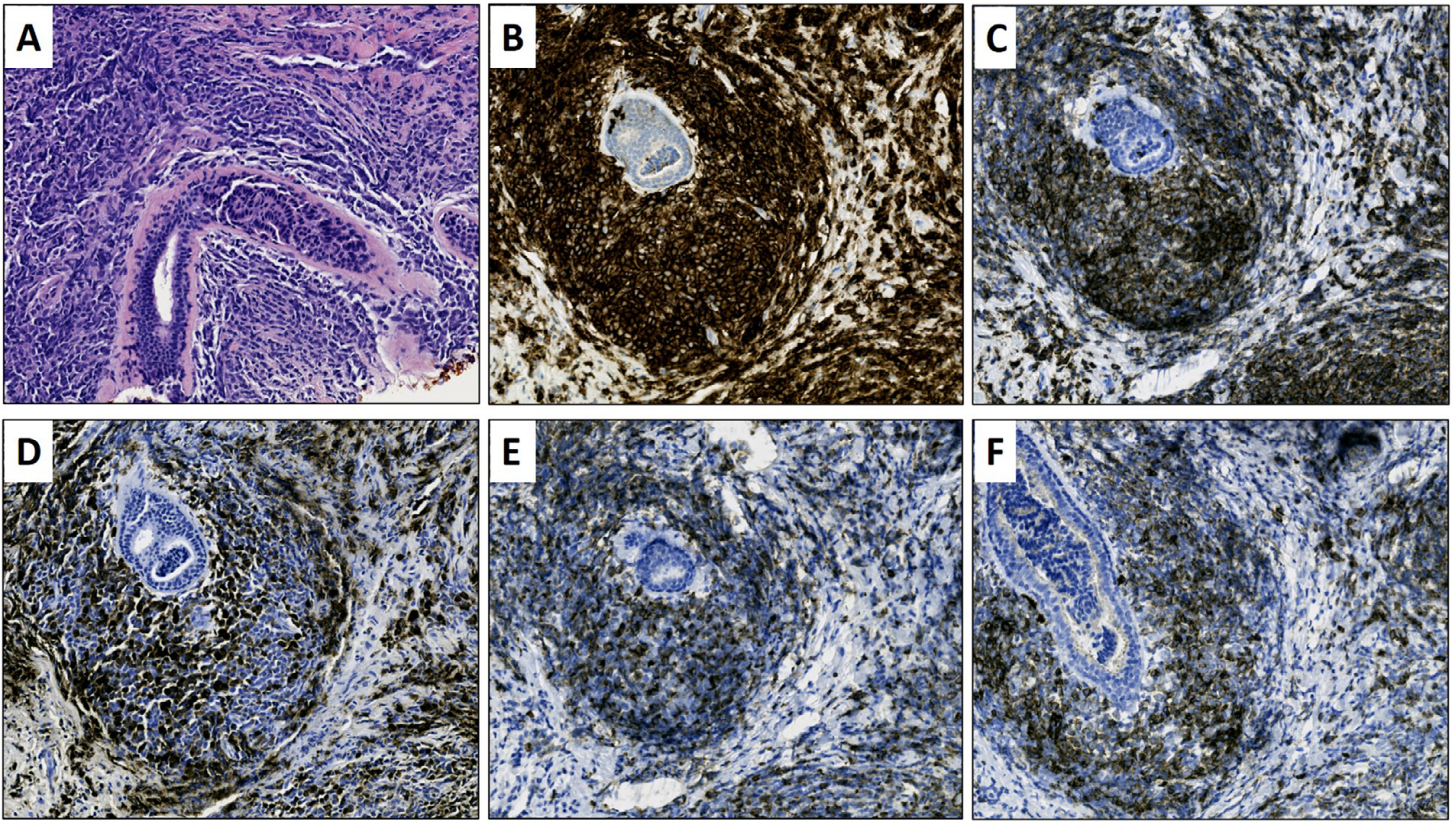
(A) H&E, 20×. Breast parenchyma is infiltrated by neoplastic cells in noncohesive, single‐file patterns, consistent with myeloid sarcoma. (B) CD45, 20×. (C) CD43, 20×. In (B) and (C), hematopoietic cells are highlighted. (D) Lysozyme, 20×. (E) MPO, 20×. (F) CD117, 20×. In (D)–(F), neoplastic cells of myeloid sarcoma are highlighted.

Breast biopsy cores showed fragments of fibroconnective tissue with atypical cellular infiltrate consisting of medium to large pleomorphic cells with vesicular chromatin, irregular nuclear contours, variable numbers of nucleoli, and acidophilic cytoplasm (Figure 1, panel A; H&E, 20×). CD45 highlighted large, pleomorphic cells surrounding breast ducts (Figure 1, panel B; 20×). CD43, lysozyme, myeloperoxidase, and CD117 were positive in the neoplastic cells (Figure 1, panels C–F; 20×). Ki‐67 proliferative index in the involved areas was approximately 95%. Pancytokeratin (AE1/AE3) was negative. After this patient's diagnosis was made, further investigation revealed an undiagnosed acute myeloid leukemia.

This report represents the identification of a rare case of myeloid sarcoma of the breast. To avoid misdiagnosis, it is important for pathologists to include myeloid sarcoma on their differential. Clinical correlation and potential evaluation for underlying hematologic malignancies are essential.

